# Fluoroscopic Findings of Extra-Cervical Facet Joint Flow and Its Incidence on Cervical Facet Joint Arthrograms

**DOI:** 10.3390/jcm9123919

**Published:** 2020-12-02

**Authors:** Hyung-Sun Won, Ho-Yeon Jang, Hyun-Seog Moon, Peng-Bo Zhu, Yeon-Dong Kim, Hyungtae Kim

**Affiliations:** 1 Department of Anatomy, Wonkwang University School of Medicine, 460 Iksan-daero, Iksan, Jeonbuk 54538, Korea; hswon01@wku.ac.kr (H.-S.W.); zhupb205@gmail.com (P.-B.Z.); 2 Jesaeng-Euise Clinical Anatomy Center, Wonkwang University School of Medicine, 460 Iksan-daero, Iksan, Jeonbuk 54538, Korea; 3 Department of Anesthesiology and Pain Medicine, Wonkwang University School of Medicine, 460 Iksan-daero, Iksan, Jeonbuk 54538, Korea; hoyunn12@naver.com; 4 Joy of the World, Interventional Pain Management Center, 10 Sinchon-ro 35-gil, Seodaemun-gu, Seoul 03774, Korea; mhspain@hanmail.net; 5 Wonkwang Institute of Science, Wonkwang University School of Medicine, 460 Iksan-daero, Iksan, Jeonbuk 54538, Korea; 6 Department of Anesthesiology and Pain Medicine, Asan Medical Center, University of Ulsan College of Medicine, 88 Olympic-ro-43-gil, Songpa-gu, Seoul 05505, Korea

**Keywords:** cervical facet joint, facet joint, facet joint block, nerve block

## Abstract

Cervical facet joint (CFJ) syndrome is a common cause of neck pain. For its diagnosis and treatment, CFJ injection with arthrogram is generally performed. This study aimed to investigate the frequency of extra-CFJ flow on CFJ arthrograms during injections and its differences according to age, sex, and cervical vertebral level. We analyzed 760 CFJ arthrograms administrated to 208 patients diagnosed with CFJ syndrome. Arthrograms at each vertebral level were collected to evaluate the normal CFJ and extra-CFJ flow. The primary and secondary outcomes were frequency of extra-CFJ flow according to cervical vertebral level, age, and sex and according to pairwise cervical levels, respectively. Extra-CFJ flow at the cervical spine occurred during 179 injections, and the overall incidence was 3.3–36.2% at different cervical levels. The incidence of extra-CFJ flow at each cervical vertebral level according to age and sex was not significant. Extra-CFJ flow was the highest at C6 and C7, but there was no statistical significance. Extra-CFJ flow was higher at lower vertebral levels (C5–C7) than at upper levels (C3 and C4). Additional clinical studies and anatomical evaluations are needed to support its clinical value and enable the development of new injection techniques.

## 1. Introduction

The prevalence of neck pain is common in the general population [1]. Neck pain can be defined as pain in the area between the skull base and the first thoracic vertebra, and may often radiate to the head or to the upper arm [2]. This kind of neck pain usually is related to nociceptive stimuli from various structures near to the vertebral column, such as muscles, ligaments including the intervertebral disc, nerve roots, and facet joints, which are often difficult to detect precisely between radicular or non-radicular pain in clinical practice.

Cervical facet joint (CFJ) syndrome is a common cause of neck and shoulder pain, and its prevalence ranges from 25 to 65% according to the criteria of the International Association for the Study of Pain [3]. CFJ syndrome is diagnosed when the following symptoms are present [4]: (1) axial neck pain; (2) pain with pressure on the spinal column at the CFJ level; (3) pain with limitations of rotation, extension, and flexion; and (4) absence of neurologic disorder.

The CFJs are innervated by the medial branches of the dorsal rami of the cervical nerves, which can be blocked by physicians for verifying the pathological origin of neck pain or treating the symptom [5,6]. Pain related to CFJ can be managed with diverse options, including medications, physical therapy, and interventional techniques, such as medial branch block and CFJ injection of local anesthetics with or without steroids [7], or radiofrequency neurotomy [8]. Many previous studies have been reported about the clinical effects of the medial branch block [9,10] and radiofrequency neurotomy [8], whereas only a few studies have attempted to reveal the CFJ injection [11].

The validity of cervical medial branch block has been generally judged through the exact needle position confirmed by contrast medium on fluoroscopy [12]. Likewise, the precise confirmation of arthrogram during a CFJ injection is essential. It may provide useful information regarding the procedure and pain relief by revealing the joint pathology and confirming the extent of the spread of injectants and involved regions, which can help localize the pain source precisely. However, no study has evaluated CFJ arthrograms according to cervical vertebral level or age group. Therefore, this study aimed to retrospectively review CFJ arthrograms in a large patient population according to cervical vertebral level and age group and analyze them by focusing on extra-CFJ flow.

## 2. Materials and Methods

This study was approved by the Institutional Review Board of Wonkwang University Hospital (IRB ID No. WKUH 2020-09-036) and was designed as a retrospective observational cohort study in a university-affiliated specialty clinic for pain management setting.

### 2.1. Participants

Data on CFJ arthrograms after CFJ injections, performed between 1 January 2018 and 31 December 2019, were collected. CFJ injections were administered to 208 patients who suffered from CFJ syndrome more than six months for both diagnostic and therapeutic purposes. A total of 760 CFJ arthrograms were obtained from the injections (C3–C7 vertebral levels). The exclusion criteria were as follows: patients with cervicogenic headache related superior to CFJ of C2 vertebral level, included into the diagnostic criteria provided by International Headache Society in 2018 [13,14]; contraindications to interventional treatment, such as pregnancy, allergy to contrast medium, coagulopathy, and infections at the injection site; insufficient needle access to the targeted CFJ due to severe anatomical deformity or a history of surgery; and unintentional intravascular or intramuscular injection. Based on age, patients were divided into two groups (≥45 years and <45 years). Table 1 shows the demographic data of the study.

### 2.2. CFJ Injections/Arthrogram Analyses

**Figure 1:**
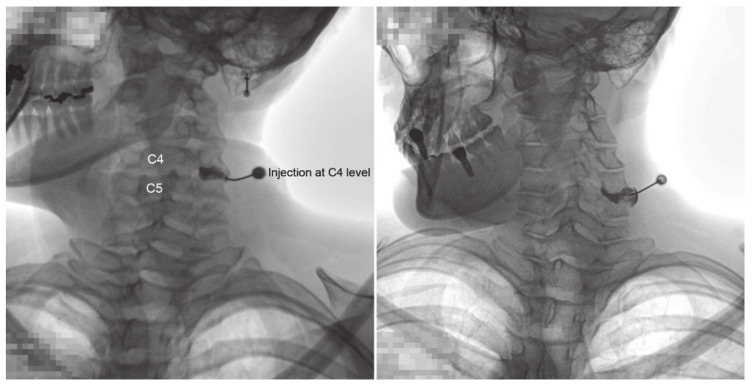
Arthrogram X-ray images showing the normal cervical facet joint flow.

**Figure 2:**
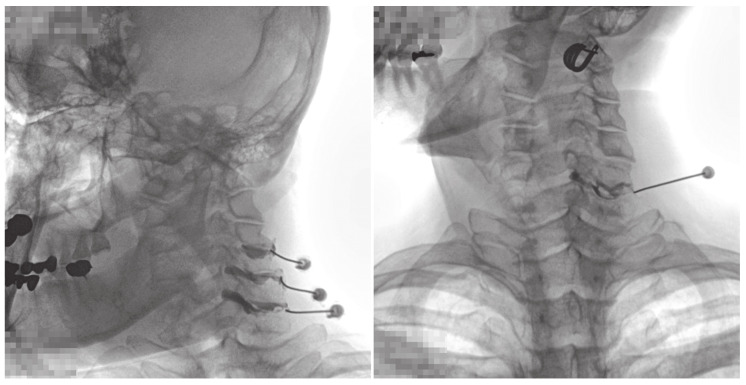
Arthrogram X-ray images showing extra-cervical facet joint flow.

All injections were administered by a pain physician, with >10 years of experience, in an operating room equipped with a fluoroscopy and an image-storing system. On the fluoroscopy table, the patient was placed in the oblique-prone position, and the skin of the posterior neck was sterilized. Fluoroscopy was performed in the anteroposterior view with the neck flexed forward. The patient’s head was slightly turned to the opposite side for appropriate visualization of the CFJ. The target CFJ level was determined based on the pain distribution in the patient. A 25-gauge, Quinke-type needle (Taechang Industrial Co., Kongju, South Korea) was inserted into the CFJ. After confirming the final position of the needle tip under fluoroscopic guidance, 0.2 mL of contrast medium (Omnipaque 300, GE Healthcare, Little Chalfont, Buckinghamshire, UK) was injected to obtain a CFJ arthrogram. Arthrograms at each vertebral level were collected to evaluate the normal CFJ and extra-CFJ flow (Figure 1 and Figure 2). Three pain physicians reviewed and verified extra-CFJ flow after a consensus discussion. If the boundary of the CFJ was seen well by contrast medium and there was no leakage to the vertebral canal, this type was normal (Figure 1). If the boundary of the CFJ was indistinct and a leakage by contrast medium was found in the vertebral canal, this type was classified as extra-CFJ flow (Figure 2). Therapeutic injections of local anesthetics with or without steroids were administered to each patient.

### 2.3. Statistical Analysis

Data regarding our study population are expressed as mean ± standard deviation or frequency (percentage). For each cervical vertebral level, the two-sample *t*-test was used for continuous variables, and the chi-square test or Fisher’s exact test was used for categorical variables. To evaluate the association between the occurrence of extra-CFJ flow and cervical vertebral level, univariate and multivariate logistic regression models were used with generalized estimating equations to account for correlations within patients. An overall *p*-value < 0.05 was considered statistically significant. In multiple comparisons between the cervical vertebral levels, statistical significance was determined with Bonferroni correction (*p*-value < 0.005). All analyses were performed using R statistical software version 3.6.3 (The R Foundation for Statistical Computing, Vienna, Austria).

## 3. Results

**Figure 3:**
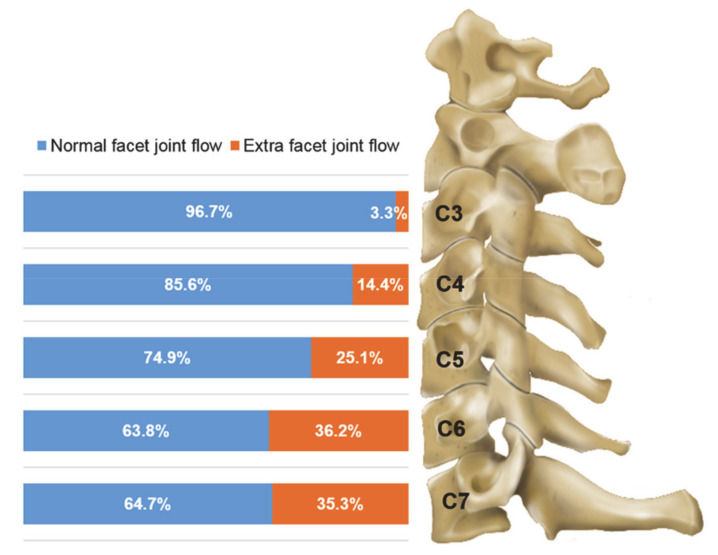
Frequency of extra-cervical facet joint flow at C3–C7 vertebral levels according to age groups and sex.

We analyzed 760 CFJ injections administered to 208 patients. Table 1 shows the demographic data of the patients. Cervical arthrograms were obtained at each cervical level and compared between the age groups and sexes (Figure 3 and Table 2). Overall, extra-CFJ flow occurred at the cervical spine during 179 of 760 (23.6%) injections, and the overall incidence was 3.3–36.2% at different cervical vertebral levels. Extra-CFJ flow was the highest at C6 (36.2%) and C7 (35.3%), but there was no statistical significance. Extra-CFJ flow was higher at lower levels (C5–C7) than at upper levels (C3 and C4). There were no statistically significant differences in age groups and sex at each cervical vertebral level.

Table 3 shows the association of occurrence of extra-CFJ flow with age, sex, and different cervical vertebral levels. The incidence of extra-CFJ flow between age groups and sex was not statistically significant. Compared to C3, the unadjusted odds ratios (ORs) were 5.51 (95% confidence interval (CI), 1.71–17.77; *p* = 0.004) for C4, 11.42 (95% CI, 3.32–39.24; *p* < 0.001) for C5, 18.77 (95% CI, 5.55–63.45; *p* < 0.001) for C6, and 19.34 (95% CI, 5.30–70.57; *p* < 0.001) for C7. Compared to C4, the unadjusted ORs were 2.07 (95% CI, 1.35–3.18; *p* = 0.001) for C5, 3.41 (95% CI, 2.18–5.32; *p* < 0.001) for C6, and 3.51 (95% CI, 1.97–6.25; *p* < 0.001). Compared to C5, the unadjusted ORs were 1.64 (95% CI, 1.14–2.37; *p* = 0.008) for C6 and 1.69 (95% CI, 0.99–2.90; *p* = 0.054) for C7. Compared to C6, the unadjusted ORs were 1.03 (95% CI, 0.62–1.72; *p* = 0.908) for C7. In general, the risk of extra-CFJ flow increased statistically significantly with decreasing levels compared to C3 and C4 after Bonferroni correction. When the lower levels were compared to C5, the increased odd ratios were not statistically significant. When C6 and C7 were compared, there were no increase in risk between those two levels. The above findings were consistent even after adjusting for age and sex for potential confounding effects in the multivariate analyses. Compared to C3, the adjusted ORs were 5.51 (95% CI, 1.73–17.55; *p* = 0.004) for C4, 11.45 (95% CI, 3.37–38.92; *p* < 0.001) for C5, 18.87 (95% CI, 5.66–62.96; *p* < 0.001) for C6, and 19.27 (95% CI, 5.34–69.48; *p* < 0.001) for C7. Compared to C4, the adjusted ORs were 2.08 (95% CI, 1.35–3.19; *p* = 0.001) for C5, 3.42 (95% CI, 2.19–5.36; *p* < 0.001) for C6, and 3.50 (95% CI, 1.96–6.24; *p* < 0.001). Compared to C5, the adjusted ORs were 1.65 (95% CI, 1.14–2.89; *p* = 0.008) for C6 and 1.68 (95% CI, 0.98–2.89; *p* = 0.059) for C7. Compared to C6, the adjusted ORs were 1.02 (95% CI, 0.61–1.71; *p* = 0.937) for C7.

## 4. Discussion

In 1983, Dory [15] first reported epidural leakage in two of 21 CFJs during steroid injections, but provided limited information for clinical practice. In later years, Manchikanti et al. tried to evaluate the adverse effects and complications of facet joint nerve block on fluoroscopic images with a greater number of patients [16]. However, they made no distinction between MBB and CFJ injection and did not investigate extra-CFJ flow. Kim et al. reported that the incidence of extra-CFJ flow during intra-articular facet joint injections was approximately 33% in the lumbar region [17]. To the best of our knowledge, studies evaluating facet joint arthrograms in the cervical region are limited compared to those in the lumbar region.

CFJ pain is defined as pain originating from the CFJ. Because the clinical symptoms [11] and imaging findings [18] of CFJ syndrome are vague and unreliable, it is clinically diagnosed by excluding other causes of cervical pain, similar to low back pain [19]. CFJ injections with hypertonic saline can reproduce similar pain in patients with neck pain and headache [20]. In this regard, Inami et al. tried to evaluate the role of intra-articular synovial folds in the cervical spine [21]. They observed the presence of putative nociceptive fibers in the cervical synovial folds, supporting the possibility of these structures as a source of CFJ pain. In addition, according to other previous studies [22,23] nociceptive innervation of the CFJ could be also observed in the joint capsule. With this background, a recently updated systematic review has shown strong evidence for the diagnostic accuracy of CFJ blocks [24]. Another study has reported strong evidence regarding the diagnostic accuracy of facet joint blocks for patients with neck pain through an evidence review [25].

The facet or zygapophyseal joint is located between the articular processes of the adjacent vertebrae. It is a synovial joint that varies in shape with the vertebral level; it is simple in the cervical and thoracic regions, but it is complex in the lumbar region. Its articular capsules are commonly thin and loose, but they seem to be more long, loose, and thin in the cervical region [26,27]. The articular capsules of each facet joint are attached to the margins of the articular processes of the adjacent vertebrae and contain some synovial fluid, which means that they prevent fluid leakage from each facet joint. In 1981, Okada first described a space communicating with the bilateral CFJs at a single vertebral level, which was regarded as a potential pathway for the spread of injectants and infection [28,29]. This potential space is also present in the lumbar region [29,30,31]. It is located between the ligamentum flavum and the vertebral arch, possibly allowing further communication with the adjacent spaces, including the interspinous space [30]. Reina et al. [31] reported that this potential space was difficult to identify anatomically because dissection could break the weak tissues included in the space, but they identified the space radiologically. Similarly, in our experience, this space has not been anatomically confirmed or detected during normal cadaveric dissection.

The facet joint has an extensive innervation of small C-type pain fibers in its synovial lining. The reactive nerve fibers and neuropeptides, such as protein gene product 9.5, substance P, and calcitonin gene-related peptide, support the evidence that the CFJ plays a key role in neck pain [21,32]. With these pain mediators, structural changes related to mechanical stress of the CFJ result from various conditions causing both acute and chronic cervical spinal pain [33]. In addition to acute injury to the joint, degenerative changes of the intervertebral disc could lead to mechanical changes in the CFJ. In other words, the loss of height of the intervertebral disc increases the load on the CFJ, which could eventually lead to CFJ degeneration [34]. A previous biomechanical study reported that peak facet joint compression with sliding and capsular ligament strains were the largest in the lower cervical vertebrae, such as C5–C6 and C6–C7, and could increase with impact acceleration [35]. Extra-CFJ flow could be observed with a relatively high frequency at the C6 and C7 vertebral levels in the present study, supporting the mechanism reported by Pearson et al. [35]. Thus, the CFJs at the C6 and C7 vertebral levels seem to be structurally vulnerable joints and have the possibility that the joint capsules in these levels are not intact unlike those of other levels.

In addition, extra-CFJ flow at these levels in the present study showed no statistical difference between the age groups. This could have a clinical implication that the vulnerability of the CFJs at the C6 and C7 vertebral levels is not directly related to the degenerative changes of these joints. To the best of our knowledge, anatomical and pathologic clinical considerations about extra-CFJ flow have not been established yet. Therefore, extra-CFJ flow should not be simply regarded as epidural leakage, as previously reported. For supplementation of the technical aspect of CFJ injections, we would like to recommend future clinical studies referring to our findings.

In a previous study, extra-facet joint flow in the lumbar region was reported as epidural leakage, which was possibly attributed to the rupture of the lumbar facet joint capsule, and there was no significant difference in the response or duration of symptom relief after lumbar facet joint injections according to the presence of epidural leakage [17]. Based on these results, Kim et al. [17] emphasized the possibility of approaching the epidural space using the transfacet approach because of the high prevalence of epidural leakage. However, the nomenclature of epidural leakage seems to be confusing because there is no anatomical confirmation in the lumbar region.

Our study has some limitations. First, more injections at the upper cervical levels, i.e., C3 and C4, are required to compare with other cervical vertebral levels, although there is a low prevalence of pathology. Second, because of the retrospective study design of the arthrogram in the medical records, patient’s previous symptoms, or pattern of extra-CFJ flow was not analyzed. In addition, we did not compare the clinical results according to extra-CFJ flow.

## 5. Conclusions

In conclusion, our study showed the actual incidence of extra-CFJ flow during fluoroscopy-guided CFJ injections. The C6 vertebral level showed a high prevalence of extra-CFJ flow. And the prevalence of extra-CFJ flow at each vertebral level was no statistical difference between the age groups and sex. In the future, additional clinical studies should be performed for its exact evaluation, and anatomical studies using cadavers are also required for to confirm the potential space related to extra-CFJ flow. These studies would enable the development of new CFJ injection techniques.

## Figures and Tables

**Table 1:** Demographic data of the study patients.

Demographics	Study Group
Number of patients	208
Sex (M/F)	75/133
Mean age (age range), years	45.7 (14–84)
Total number of injections	760

M, male; F, female.

**Table 2:** Summary statistics of study population.

Cervical Level	Extra-CFJ Flow (*n*)	Age (mean ± SD)	Age Categories (*n*)		Sex (*n*)		Number of Injection (*n*)
			<45 Years	≥45 Years	Female	Male	
C3	No (89)	46.8 ± 13.72	42	47	57	32	92
	Yes (3)	50.33 ± 4.04	0	3	2	1	
	*p-*value	0.664	0.247		1.000		
C4	No (166)	45.53 ± 12.52	80	86	101	65	194
	Yes (28)	49.14 ± 13.42	11	17	22	6	
	*p-*value	0.164	0.419		0.072		
C5	No (155)	45.44 ± 12.83	77	78	99	56	207
	Yes (52)	46.54 ± 11.97	23	29	33	19	
	*p-*value	0.587	0.525		0.958		
C6	No (127)	44.86 ± 13.00	66	61	82	45	199
	Yes (72)	47.61 ± 11.68	28	44	43	29	
	*p-*value	0.138	0.079		0.497		
C7	No (44)	46.02 ± 12.71	20	24	28	16	68
	Yes (24)	44.92 ± 12.98	12	12	13	11	
	*p-*value	0.735	0.802		0.446		

CFJ, cervical facet joint; SD, standard deviation. Two-sample *t*-test was used for continuous variables. Chi-square test or Fisher’s exact test was used for categorical variables. Age categories were determined based on the mean value.

**Table 3:** Univariate and multivariate logistic regression analyses of extra-cervical facet joint flow occurrence comparing pairwise cervical levels.

Variable	Category	Unadjusted OR (95% CI)	*p-*Value	Adjusted OR (95% CI)	*p-*Value
**Age**	<45 years	Reference		Reference	
	≥45 years	1.35 (0.90–2.02)	0.15	1.37 (0.89–2.89)	0.152
**Sex**	Female	Reference		Reference	
	Male	1.04 (0.69–1.57)	0.845	1.05 (0.68–1.62)	0.825
**Cervical level**	C4 vs. C3	5.51 (1.71–17.77)	0.004*	5.51 (1.73–17.55)	0.004*
	C5 vs. C3	11.42 (3.32–39.24)	<0.001*	11.45 (3.37–38.92)	<0.001*
	C6 vs. C3	18.77 (5.55–63.45)	<0.001*	18.87 (5.66–62.96)	<0.001*
	C7 vs. C3	19.34 (5.30–70.57)	<0.001*	19.27 (5.34–69.48)	<0.001*
	C5 vs. C4	2.07 (1.35–3.18)	0.001*	2.08 (1.35–3.19)	0.001*
	C6 vs. C4	3.41 (2.18–5.32)	<0.001*	3.42 (2.19–5.36)	<0.001*
	C7 vs. C4	3.51 (1.97–6.25)	<0.001*	3.50 (2.19–6.24)	<0.001*
	C6 vs. C5	1.64 (1.14–2.37)	0.008	1.65 (1.14–2.38)	0.008
	C7 vs. C5	1.69 (0.99–2.90)	0.054	1.68 (0.98–2.89)	0.059
	C7 vs. C6	1.03 (0.62–1.72)	0.908	1.02 (0.61–1.71)	0.937

OR, odds ratio; CI, confidence interval. Adjusted for age and sex. * Reached statistical significance with Bonferroni correction (*p*-value < 0.005).

## References

[1] Côté P., Cassidy J.D., Carroll L.J., Kristman V. (2004). The annual incidence and course of neck pain in the general population: A population-based cohort study. Pain.

[2] Guzman J., Hurwitz E.L., Carroll L.J., Haldeman S., Côté P., Carragee E.J., Peloso P.M., van der Velde G., Holm L.W., Hogg-Johnson S. (2008). A new conceptual model of neck pain: Linking onset, course, and care: The bone and joint decade 2000-2010 task force on neck pain and its associated disorders. Spine (Phila Pa. 1976).

[3] Manchikanti L., Singh V., Rivera J., Pampati V. (2002). Prevalence of cervical facet joint pain in chronic neck pain. Pain Physician.

[4] Van Eerd M., Patijn J., Lataster A., Rosenquist R.W., van Kleef M., Mekhail N., Van Zundert J. (2010). 5. Cervical facet pain. Pain Pract..

[5] Barnsley L., Lord S., Wallis B., Bogduk N. (1993). False-positive rates of cervical zygapophysial joint blocks. Clin. J. Pain.

[6] Jeon Y.H., Kim S.Y. (2015). Detection rate of intravascular injections during cervical medial branch blocks: A comparison of digital subtraction angiography and static images from conventional fluoroscopy. Korean J. Pain.

[7] Lord S.M., Barnsley L., Bogduk N. (1995). The utility of comparative local anesthetic blocks versus placebo-controlled blocks for the diagnosis of cervical zygapophysial joint pain. Clin. J. Pain.

[8] Lord S.M., Barnsley B.J., Wallis B.J., McDonald G.J., Bogbuk N. (1996). Percutaneous radio-frequency neurotomy for chronic cervical zygapophyseal-joint pain. N. Engl. J. Med..

[9] Manchikanti L., Manchikanti K.N., Damron K.S., Pampati V. (2004). Effectiveness of cervical medial branch blocks in chronic neck pain: A prospective outcome study. Pain Physician.

[10] Manchikanti L., Singh V., Falco F.J., Cash K.A., Fellows B. (2010). Comparative outcomes of a 2-year follow-up of cervical medial branch blocks in management of chronic neck pain: A randomized, double-blind controlled trial. Pain Physician.

[11] Bogduk N., Marsland A. (1988). The cervical zygapophysial joints as a source of neck pain. Spine (Phila Pa. 1976).

[12] Barnsley L., Bogduk N. (1993). Medial branch blocks are specific for the diagnosis of cervical zygapophyseal joint pain. Reg. Anesth..

[13] Headache Classification Committee of the International Headache Society (HIS) (2018). The international classification of headache disorders, 3rd edition. Cephalalgia.

[14] Wang L., Shen J., Das S., Yang H. (2020). Diffusion tensor imaging of the C1-C3 dorsal root ganglia and greater occipital nerve for cervicogenic headache. Korean J. Pain.

[15] Dory M.A. (1983). Arthrography of the cervical facet joints. Radiology.

[16] Manchikanti L., Malla Y., Wargo B.W., Cash K.A., Pampati V., Fellows B. (2012). Complications of fluoroscopically directed facet joint nerve blocks: A prospective evaluation of 7500 episodes with 43,000 nerve blocks. Pain Physician.

[17] Kim S., Lee J., Chai J., Lee G., You J., Kang H., Ahn J. (2015). Fluoroscopy-guided intra-articular facet joint steroid injection for the management of low back pain: therapeutic effectiveness and arthrographic pattern. J. Korean Soc. Radiol..

[18] Farrell S.F., Smith A.D., Hancock M.J., Webb A.L., Sterling M. (2019). Cervical spine findings on MRI in people with neck pain compared with pain-free controls: A systematic review and meta-analysis. J. Magn. Reson. Imaging.

[19] Schwarzer A.C., Wang S.C., O’Driscoll D., Harrington T., Bogduk N., Laurent R. (1995). The ability of computed tomography to identify a painful zygapophysial joint in patients with chronic low back pain. Spine (Phila Pa. 1976).

[20] Pawl R.P. (1977). Headache, cervical spondylosis, and anterior cervical fusion. Surg. Ann..

[21] Inami S., Shiga T., Tsujino A., Yabuki T., Okado N., Ochiai N. (2001). Immunohistochemical demonstration of nerve fibers in the synovial fold of the human cervical facet joint. J. Orthop. Res..

[22] Kallakuri S., Singh A., Chen C., Cavanaugh J.M. (2004). Demonstration of substance P, calcitonin gene-related peptide, and protein gene product 9.5 containing nerve fibers in human cervical facet joint capsules. Spine (Phila Pa. 1976).

[23] Farrell S.F., Osmotherly P.G., Cornwall J., Rivett D.A. (2016). Immunohistochemical investigation of nerve fiber presence and morphology in elderly cervical spine meniscoids. Spine J..

[24] Falco F.J., Manchikanti L., Datta S., Wargo B.W., Geffert S., Bryce D.A., Atluri S., Singh V., Benyamin R.M., Sehgal N. (2012). Systematic review of the therapeutic effectiveness of cervical facet joint interventions: An update. Pain Phys..

[25] Rubinstein S.M., van Tulder M. (2008). A best-evidence review of diagnostic procedures for neck and low-back pain. Best Pract. Res. Clin. Rheumatol..

[26] Woodburne R., Burkel W. (1994). Essentials of human anatomy.

[27] Soames R., Williams P., Bannister L., Berry M., Collins P., Dyson M., Dussek J., Ferguson M. (1995). Skeletal system. Gray’s Anatomy : The Anatomical Basis of Medicine and Surgery.

[28] Chen C.K., Yeh L., Resnick D., Lai P.H., Liang H.L., Pan H.B., Yang C.F. (2004). Intraspinal posterior epidural cysts associated with Baastrup’s disease: Report of 10 patients. AJR Am. J. Roentgenol..

[29] Lehman V.T., Murthy N.S., Diehn F.E., Verdoorn J.T., Maus T.P. (2015). The posterior ligamentous complex inflammatory syndrome: Spread of fluid and inflammation in the retrodural space of Okada. Clin. Radiol..

[30] Thorpe Lowis C.G., Xu Z., Zhang M. (2018). Visualisation of facet joint recesses of the cadaveric spine: A micro-CT and sheet plastination study. BMJ Open Sport Exerc. Med..

[31] Reina M.A., Avellanal M., Boezaart A.P., Tubbs R.S., De Andrés J., Nin O.C., Prats-Galino A. (2020). Case series of fluoroscopic findings and 3D reconstruction of human spinal MRIs of the space of Okada. Clin. Anat..

[32] Kim K.H., Choi S.H., Kim T.K., Shin S.W., Kim C.H., Kim J.I. (2005). Cervical facet joint injections in the neck and shoulder pain. J. Korean Med. Sci..

[33] Manchikanti L. (1999). Facet joint pain and the role of neural blockade in its management. Curr. Rev. Pain.

[34] Kirpalani D., Mitra R. (2008). Cervical facet joint dysfunction: A review. Arch. Phys. Med. Rehabil..

[35] Pearson A.M., Ivancic P.C., Ito S., Panjabi M.M. (2004). Facet joint kinematics and injury mechanisms during simulated whiplash. Spine.

